# Do antipsychotics limit disability in schizophrenia? A naturalistic comparative study in the community

**DOI:** 10.4103/0019-5545.58893

**Published:** 2010

**Authors:** Jagadisha Thirthalli, Basappa K. Venkatesh, Magadi N. Naveen, Ganesan Venkatasubramanian, Udupi Arunachala, Kengeri V. Kishore Kumar, Bangalore N. Gangadhar

**Affiliations:** Department of Psychiatry, National Institute of Mental Health And Neurosciences (NIMHANS), Bangalore, India; 1 Manasa Nursing Home, Thirthahalli-577432, Shimoga District, Karnataka, India

**Keywords:** Schizophrenia, antipsychotics, disability

## Abstract

**Background:**

Though antipsychotics are effective against symptoms of schizophrenia and prevent relapses, their effect on disability has not been studied in a comparative design.

**Aim:**

To compare disability of schizophrenia patients receiving continuous antipsychotic treatment with that of those not receiving or receiving irregular treatment in a rural community setting using a naturalistic comparative study design.

**Patients and Methods:**

Disability was assessed in 182 schizophrenia patients living in Thirthahalli Taluk of Shimoga District, Karnataka, using Indian Disability Evaluation and Assessment Scale (IDEAS). Fifty patients (27.5%) were receiving regular treatment in the previous 2 years and their disability was assessed for the period when they were on antipsychotics. The remaining 132 patients (72.5%) had off-antipsychotics periods in the previous 2 years and their disability was assessed for the period when they were off-antipsychotics.

**Results:**

Patients on antipsychotics had significantly less disability across all domains of disability and in total IDEAS scores. Multivariate regression analysis showed that treatment status predicted disability scores after controlling for the effects of the confounding factors. Different levels of exposure to antipsychotic treatment were associated with different levels of disability.

**Conclusions:**

Treatment with antipsychotics is associated with significantly less disability. There is an urgent need to bring schizophrenia patients under the umbrella of treatment.

## INTRODUCTION

Antipsychotics have been the mainstay treatment modality in treating schizophrenia; they cause reduction of symptoms and prevent relapses in most schizophrenia patients. However, it is still not clear if they make any meaningful difference to the lives of the sufferers, there is only a modest correlation between the severity of symptoms and disability in schizophrenia.[1–3] Antipsychotics are particularly effective against positive symptoms, but these symptoms are poorly correlated with functional outcome.[4,5] In spite of the use of antipsychotic drugs for the past five decades, schizophrenia has remained one of the most disabling conditions. It is ranked the ninth leading cause of disability among all the disorders causing disability in the world.[6] Many authors have stressed the need to focus beyond just the symptom reduction.[7,8] Disability is an important outcome that is meaningful for the patients and their family members. Implementation of the Persons with Disabilities Act (PDA) 1995 has enhanced the importance of this outcome dimension.

Do antipsychotics limit disability in schizophrenia? Schizophrenia has a chronic, deteriorating course if untreated. Does treatment with antipsychotics change this course? Existing literature alludes to this question only tangentially. For example, Hegarty *et al*.[9] in their meta-analysis of outcome studies spanning over 100 years observed that the proportion of patients with poor outcome has decreased after the advent of antipsychotic drugs, but a direct causal role of antipsychotics cannot be inferred from this. Many authors have observed that early treatment with antipsychotics is associated with better functioning.[10,13] However, there are contradictory findings too.[14,15] Some skeptics have even criticized the role of antipsychotics for their potential harm on the course of the disease.[16] Recent editorials in leading journals have been tentative about their help to schizophrenia patients.[17]

The best way to answer the question as to whether antipsychotic treatment causes better functional outcome is to conduct large-scale, long-term placebo controlled studies,[18] which at this stage are not possible on ethical grounds. Tandon[19] observes that with the gold standard method not feasible, an in-depth analysis of existing data is the only way out to answer this important question. In any community in India, a proportion of schizophrenia patients are expected to live without exposure to antipsychotic treatment and they form a naturalistic comparison group to assess if those exposed to antipsychotic drugs have less disability. This paper makes use of the data obtained in one such naturalistic experiment. Here, we compare the disability of schizophrenia patients receiving continuous antipsychotic medications with that of those having exposure to different levels of treatment, including no treatment.

## PATIENTS AND METHODS

Subjects: Sample for this study included schizophrenia patients recruited for the Community Intervention in Psychotic Disorders (CoInPsyD) project in Thirthahalli taluk of Shimoga District of Karnataka. The project entails identifying all schizophrenia patients living in this rural community, treating and following them up. Fifty-four rural health workers were trained in identifying patients with severe mental disorders in the community and were asked to refer all such patients irrespective of their treatment status. In addition, two trained social workers interviewed the health workers about the presence of persons with symptoms of psychosis in each family under their care referring to their community survey registers (a total of 29 432 families for the entire community of 1 43 345 population). A research psychiatrist interviewed all patients, thus identified and diagnoses were assigned using the ICD-10-Diagnostic Criteria for Research (ICD-10-DCR[20]). A senior psychiatrist confirmed the diagnosis of schizophrenia after an independent clinical interview. A total of 207 persons were diagnosed as having schizophrenia. The health workers reported to the study team about the presence, in the community, of 20 other persons with features suggesting schizophrenia. These could not be interviewed because of several reasons including refusal to give consent, being severely ill with no caretakers to give any information, etc. Among the 207, we could reliably assess the treatment history and disability in 182 (88%) persons (94 males; 51.4%). These form the sample for this study.

### Assessments

**Figure 1:**
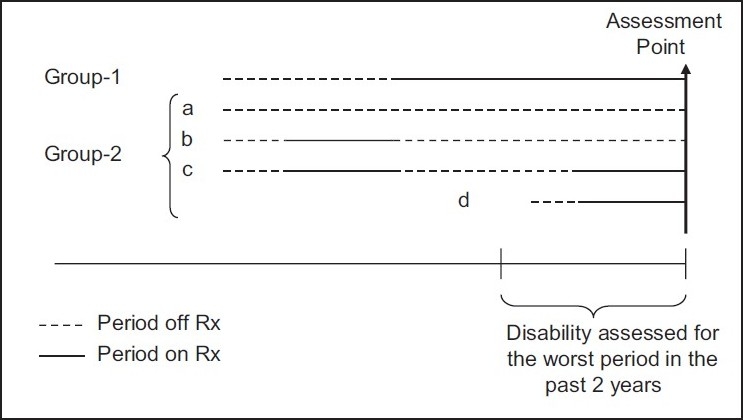
Pictorial depiction of the relationship between treatment status and period of disability assessment: Group 1: Those who were on regular treatment with antipsychotics during the entire period of last 2 years (n = 50): Group 2: Those who were not on treatment for sometime in the past 2 years (Group 2; n = 132): (a) patients who were never treated (n = 30), (b) those who were treated at sometime during their lifetime and then stopped it (n = 49) and (c) those who had stopped antipsychotics and had restarted them because of exacerbation of symptoms (n = 45) (d) those whose psychosis started within the past 2 years (n = 8)

1. Disability: Indian Disability Evaluation and Assessment Scale (IDEAS[21]) was used to assess the level of disability. A trained psychiatrist assessed their disability. Information for this was gathered from the patients, their family members, and health workers. They were asked to give information about the patient's functioning during the worst part of his/her illness in the previous 2 years. Disability across four domains of self-care (SCR), interpersonal relationship (IPR), communication and understanding (COM), and work (WRK) was scored based on this information.
  IDEAS is originally developed for measuring and certifying disability for psychiatric patients in India. In this, disability is scored from 0 to 4 for each item and the sum of the four item scores gives the total disability score. Global disability score is calculated by adding “total disability score” and a score for the duration of illness (DOI score). For the purposes of this study, only total disability score was analyzed. This was done because we wanted to focus on the current level (past 2 years) of disability irrespective of the total duration of illness. IDEAS has satisfactory face, content and criterion validity, and internal consistency. It has also been recently used for research purposes satisfactorily.[22]
  The research psychiatrist was trained in administering IDEAS at National Institute of Mental Health & Neurosciences (NIMHANS), Bangalore. Inter-rater reliability of IDEAS was assessed on 10 patients with schizophrenia at NIMHANS (these patients were not part of the study). Intra-class correlation for the total IDEAS scores was excellent (0.976).
2. Treatment history: Patients and the family members were asked to provide all information including medical records pertaining to patients' mental illness. The patients were divided into two groups [Figure 1]:
  1. those who were on regular treatment with antipsychotics during the entire period of last 2 years (Group 1; n = 50) and
  2. those who were not on treatment for sometime in the past 2 years (Group 2; n = 132). This group consisted of
    - patients who were never treated (n = 30)
    - those who were treated at sometime during their lifetime and then stopped it (n = 49)
    - those who had stopped antipsychotics and had restarted them because of exacerbation of symptoms (n = 45) and
    - those whose psychosis started within the past 2 years (n = 8).

In those who had some period off treatment in the past 2 years the patients and the family members recognized the off-treatment period as the worst period. Thus, in Group 1, disability was assessed for the period while patients were on antipsychotics, and in Group-2, it was assessed for the period while patients were off-antipsychotics. Two social workers collected the clinical and socio-demographic details of the subjects and this included treatment history. There was an overall agreement between the assessment done by the psychiatrist and the social workers. None of the patients in either group was receiving any structured psychosocial interventions.

### Statistical analysis

Group-1 and Group-2 were compared using independent-sample *t*-test and Chi-square test for continuous and categorical variables respectively. Analysis of Variance (ANOVA) with Scheffe's *post-hoc* test was used to compare the disability scores across the subgroups. Finally, multiple linear regression analysis was used to examine the relationship between disability scores and treatment status, controlling for the potential confounding effects of other predictor variables.

## RESULTS

The mean age (SD) of the subjects was 41.3 (11.0) years; their mean age at the onset (SD) of psychosis was 29.3 (10.4) years; they were ill for a mean period (SD) of 11.3 (8.8) years; the mean years of education (SD) was 6.54 (4.7). Most (48.3%) belonged to the lower socioeconomic status. About 18% had comorbid alcohol dependence/harmful use. Table 1 shows the comparison between the two groups for clinical and sociodemographic parameters. There was no statistically significant difference in any parameters.

**Table 1:** Differences between the two groups 1 and 2

Variables	Groups		*t*/Chi-square test	*P* value
			
	Group-1 (n = 50)	Group-2 (n = 132)		
Age (years)	43.6 (11.3)	40.4 (10.8)	1.73	0.085
Sex (males)*	29 (58)	65 (48.9)	1.21	0.271
Age at the onset (years)	30.9 (9.7)	29.6 (10.7)	0.74	0.46
Duration of illness (years)	12.7 (6.9)	10.8 (9.3)	1.29	0.199
Socio-economic status*				
Lower	21 (42.4)	67 (50.6)	1.42	0.496
Middle	17 (33.3)	45 (34.1)		
Upper	12 (24.2)	20 (15.3)		
Years of education	6.79 (4.7)	6.44 (4.8)	0.43	0.664
Alcohol abuse/dependence*	9 (18)	23 (17.4)	0.008	0.927

* There were no differences between the two groups in any of the parameters studied; Figures are in Mean (SD) except for those with, where the figures are absolute numbers (percentages)

**Figure 2:**
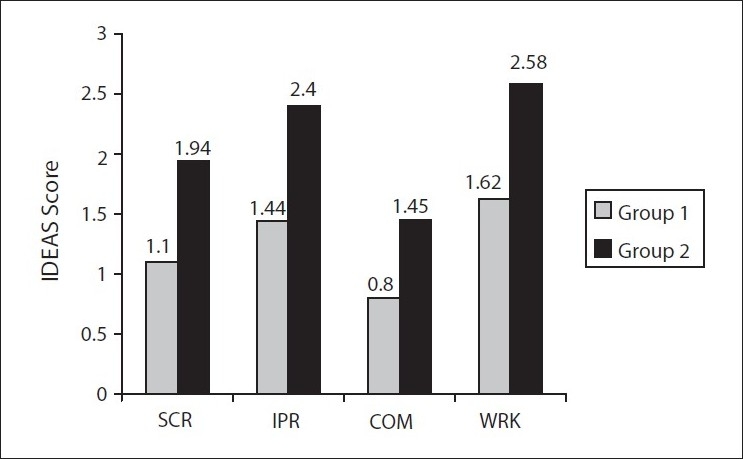
Comparison of IDEAS scores across the four domains. All comparisons were significant at *P* < 0.001

Figure 2 shows the difference between the two groups on disability in the four domains. The disability was significantly less in Group 1 than in Group 2 across all the domains (*P* < 0.001). The mean total disability (SD) in Group 1 was 5.0 (4.0) and in Group 2 was 8.3 (3.8); (T = 5.14; *P* < 0.001). A cut-off of 40% disability was considered to categorize patients as being disabled or not. Forty percent of the patients in Group 1 were disabled in contrast to Group 2, in which 71% were disabled (chi-square = 14.6; *P* < 0.001). The odd ratio was 0.277 (95% CI: 0.14-0.55), suggesting that subjects in Group 1 had about one-third the risk of suffering disability compared to those in Group 2.

**Figure 3:**
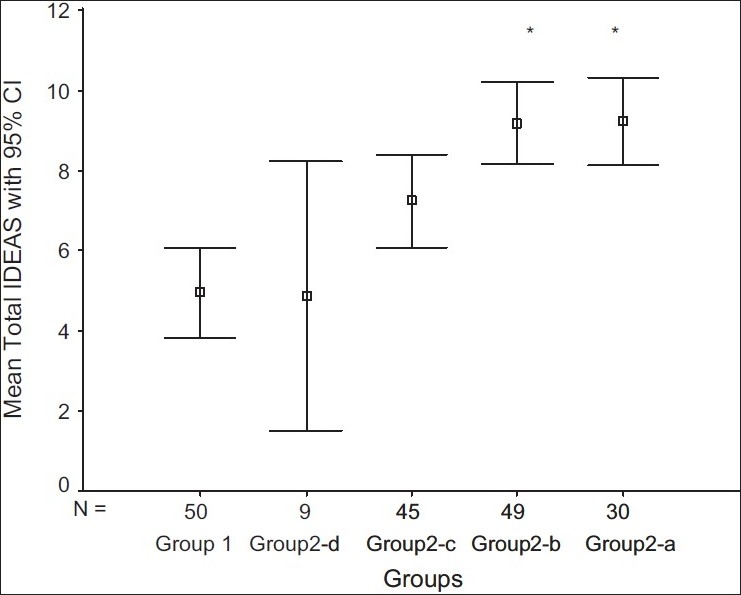
Comparison of total IDEAS score across the groups:* Scheffe's post hoc test: Significantly greater than Group 1 (*P* < 0.001)

One-way analysis of variance (ANOVA) was performed to compare the IDEAS total score across the groups. The groups were significantly different in IDEAS total score (model F = 10.72; df = 4, 177; *P* < 0.001). Scheffe's *post hoc* analysis showed that Group 1 differed significantly from Group 2(a) (mean difference = 4.27; SE = 0.86; *P* < 0.001) and Group 2(b) (mean difference = 4.22; SE = 0.75; *P* < 0.001). The other two subgroups of Group 2 were comparable to Group 1 in total IDEAS score [Figure 3].

Linear regression analysis was performed with total IDEAS score as the dependent variable and treatment status, age, sex, education, socioeconomic status, duration of symptoms, and alcohol dependence/harmful use as predictors. The model was significant at *P* < 0.001 and explained 14% of the variance. Again, the treatment status emerged as a significant predictor (B = 3.27; SE = 0.96; *P* < 0.001) and this alone explained 12% of the variance.

## DISCUSSION

The most important finding of this study was that being on antipsychotic treatment was associated with significantly less disability. Being off-treatment was associated with about three times higher risk of suffering disability. Different levels of treatment exposure were associated with different levels of disability, thus suggesting a “dose-dependent” effect of antipsychotic treatment in schizophrenia.

The strengths of this study are that it was conducted with a naturalistic design in a community. Hence, patients from the same background, who differed on their antipsychotic treatment, could be compared. That the two groups were “comparable” is borne by the fact that they did not differ in any of the clinical or socio-demographic variables. A hospital-based study of this nature would have limited applications, as only a small biased sample would reach hospitals. The exhaustive key-informant survey tapped all consenting patients in the community. The point prevalence of schizophrenia in this study was 1.6 per thousand (age unadjusted; 95% CI 1.4 - 1.8). Similar prevalence figures have been reported for schizophrenia in rural communities of India[23] and Sri Lanka.[24] This validates the exhaustiveness of the gathering the community sample. A trained psychiatrist who demonstrated excellent inter-rater reliability with a senior psychiatrist administered IDEAS. This instrument is the most relevant one for assessing psychiatric disability among Indian patients. It is the official tool to assess disability in India and hence is widely used.

Is the difference in disability attributable to antipsychotic treatment? Being a naturalistic comparative study, the groups were not randomized. We examined, using multivariate regression analysis, the effects of possible confounding factors including age, sex, duration of symptoms, education, socio-economic status, and alcohol dependence/harmful use and the treatment status still emerged as significant predictor of disability. Since no subject was on any structured psychosocial intervention or rehabilitation programe, these factors cannot account for the difference in disability. However, we did not assess the effects of other confounding factors including expressed emotions, family support, etc., which might have played a role in influencing disability. For instance, it is possible that those who were regular on treatment did so solely because of good family support, and such families might have encouraged the patients in such ways as to reduce their disability. On the other hand, those who were never treated may have had families, which were negligent of the patients, and their disability may be reflective of the family pathology and not so much of the lack of antipsychotic use. One may still conclude that “treatment” and not just “antipsychotic drugs” is associated with less disability.

To our knowledge, this is the first study to use such a design to assess outcome of schizophrenia. The findings of this study are in keeping with the clinical lore that antipsychotic treatment is helpful and also with the literature that has shown association between compliance with antipsychotic treatment and disability.[2,25,26] However, none of the past studies directly compared patients on treatment with those not on treatment for the assessment of their disability.

Assessment of disability using IDEAS meant that the disability was assessed for the worst period in the previous 2 years. Therefore, it was not possible to assess the patient's psychopathology for that period and hence its influence on disability could not be studied.

Raters, who were not blind to the group status of the patients, assessed disability. This paper is a secondary analysis of the data of a project, which has a different hypothesis. Disability was assessed as part of the baseline status of the subjects. The rater did not have any plans of analyzing the data in this way. In fact, the groups were formed as a *post hoc* exercise and not as a *priory* groups. Therefore, it is unlikely that lack of “blinding” would have influenced the results. Not using a standardized method to collect treatment details remains a limitation of the study.

In summary, being on antipsychotic treatment was associated with least disability. Over 70% of schizophrenia patients in the community have been denied treatment in the developed countries.[27] There is an urgent need for public health initiative to bring all such patients under treatment umbrella.
